# Multiple *Anopheles* species complicate downstream analysis and decision-making in a malaria pre-elimination area in southern Mozambique

**DOI:** 10.1186/s12936-024-04842-0

**Published:** 2024-01-18

**Authors:** Mara Máquina, Mercy A. Opiyo, Nelson Cuamba, Dulcisária Marrenjo, Maria Rodrigues, Sarmento Armando, Sheila Nhate, Fabião Luis, Francisco Saúte, Baltazar Candrinho, Neil F. Lobo, Krijn P. Paaijmans

**Affiliations:** 1 Centro de Investigação em Saúde de Manhiça (CISM), Fundação Manhiça, Manhica, Mozambique; 2 ISGlobal, Barcelona, Spain; 3 Malaria Elimination Initiative, University of California San Francisco, San Francisco, CA USA; 4 Programa Nacional de Controlo da Malária, Ministério da Saúde, Maputo, Mozambique; 5 PMI VectorLink Project, Abt Associates Inc., Maputo, Mozambique; 6 Malaria Consortium, Maputo, Mozambique; 7 Maputo Provincial Health Service, Matola, Mozambique; 8 Eck Institute for Global Health, University of Notre Dame, Notre Dame, IN USA; 9 Center for Evolution and Medicine, School of Life Sciences, Arizona State University, Tempe, AZ USA; 10 Simon A. Levin Mathematical, Computational and Modeling Sciences Center, Arizona State University, Tempe, AZ USA

## Abstract

**Background:**

Different anopheline species (even within a species group/complex) can differ in their feeding and resting behaviours, which impact both malaria transmission patterns as well as the efficacy of vector control interventions. While morphological identification of sampled specimens is an important first step towards understanding species diversity and abundance, misidentification can result in the implementation of less effective vector control measures, and consequently smaller reductions in the number of local malaria cases. Focusing on southern Mozambique, a malaria pre-elimination area where malaria remains persistent, the aims of this preliminary study were to use molecular identification (CO1 and ITS2 barcoding) to (1) validate the results from the morphological identification (with a particular focus on *Anopheles pharoensis* and *Anopheles squamosus*), and (2) have a closer look at the *Anopheles coustani* group (which includes *Anopheles tenebrosus* and *Anopheles ziemanni*).

**Methods:**

Female anopheline mosquitoes (*n* = 81) were identified morphologically and subsequently sequenced at the ribosomal DNA internal transcribed spacer region 2 (ITS2) and/or cytochrome oxidase subunit 1 (CO1) loci towards species determination.

**Results:**

Out of the 62 specimens that were identified morphologically to species, 4 (6.5%) were misidentified. Regarding the *An. coustani* group, morphological identification showed that several members are present in southern Mozambique, including *An. coustani* sensu lato (s.l.), *An. ziemanni* and *An. tenebrosus*. However, based on both ITS2 and CO1 sequences, the exact species remains unknown for the latter two members until voucher sequences are available for comparison.

**Conclusion:**

The reason(s) for morphological misidentification of anopheline mosquitoes need to be mitigated. This is usually related to both the capacity (i.e. training) of the microscopist to identify anopheline species, and the information provided in the dichotomous identification key. As the *An. coustani* complex contributes to (residual) malaria transmission in sub-Saharan Africa, it may play a role in the observed persistent malaria in southern Mozambique. A better baseline characterizing of the local anophelines species diversity and behaviours will allow us to improve entomological surveillance strategies, better understand the impact of vector control on each local vector species, and identify new approaches to target those vector species.

**Supplementary Information:**

The online version contains supplementary material available at 10.1186/s12936-024-04842-0.

## Background

Mozambique is working collaboratively with South Africa and Eswatini to move both South Africa and Eswatini to elimination, and move southern Mozambique to pre-elimination. The MOSASWA initiative supports these goals at sub-regional and in-country transmission areas, with indoor residual spraying (IRS) as the main vector control intervention at the district level [1, 2], on top of long-lasting insecticidal nets (LLINs) that are distributed by the country.

Historically, *Anopheles funestus* sensu stricto (s.s.) has been the major malaria vector in southern Mozambique [3–5], with *Anopheles arabiensis* and *Anopheles merus* (sibling species within the *Anopheles gambiae* species complex) playing a minor role in malaria transmission [6–8]. However, the selection pressure by IRS and LLINs on mosquito vector populations over recent decades, and the last decade in particular, have resulted in a change in (1) species compositions within the *Anopheles funestus* group (i.e. reductions in the proportion *An. funestus* s.s. and increases in *Anopheles leesoni* and *Anopheles parensis* proportions [6, 8]), and (2) the relative importance of *An. funestus* s.s. in regional malaria transmission [6, 8, 9]. Other anopheline mosquitoes have now been incriminated as malaria vectors in Mozambique’s southern provinces, including *Anopheles squamosus* [8], a member of the *An. funestus* group: *An. parensis* [8], and a member of the *Anopheles coustani* group: *Anopheles tenebrosus* [3, 9]. Other species that are frequently collected—but not yet found to be malaria positive [8, 9]—are known malaria vectors elsewhere in sub-Saharan Africa. These include *Anopheles rufipes* [10], *Anopheles pharoensis* [11], two members of the *An. funestus* species group: *Anopheles rivulorum* [12] and *An. leesoni* [13], *An. coustani* sensu lato (s.l.) [14, 15] and a particular member of this *An. coustani* group: *Anopheles ziemanni* [15, 16].

Accurate species identification is important, as different anopheline species (even within a species group/complex) have variable feeding and resting behaviours. This consequently affects spatial and temporal malaria transmission, and the efficacy of vector control interventions [17, 18]. Morphological identification of sampled specimens is an important first step towards understanding species diversity [19]. Molecular diagnostic PCR assays can further differentiate members of the *An. funestus* group and *An. gambiae* complex [20, 21]. Further molecular identification [e.g., sequencing of the mitochondrial DNA cytochrome *c* oxidase subunit I (CO1) and/or ribosomal internal transcribed spacer region 2 (ITS2)] may be warranted to validate morphological identification and identify species not captured in the diagnostic assays [22, 23]. This was recently highlighted for *Anopheles namibiensis* in Mopeia District (Zambezia Province, central Mozambique), which was morphologically identified as *An. tenebrosus* [24].

As southern Mozambique moves towards pre-elimination, accurate species identification is a prerequisite for effective vector control decision-making with expected impacts on species compositions and bionomic traits [19]. The aims of this preliminary study were to use molecular identification (CO1 and ITS2 barcoding) to (1) validate the results from the morphological identification (with a particular focus on *An. pharoensis* and *An. squamosus*), and (2) have a closer look at the *An. coustani* group (which includes *An. tenebrosus* and *An. ziemanni*).

## Methods

Female anopheline mosquitoes (*n* = 81) were selected from two study areas (Matutuine district and Manhiça village, both in Maputo province) for further molecular analysis. Both areas experience persistencet malaria transmission, despite prompt diagnosis and effective treatment of confirmed malaria cases, IRS and LLINs [25, 26]. The sample included randomly selected *An. pharoensis* (*n* = 22), *An. tenebrosus* (*n* = 14), *An. ziemanni* (*n* = 16), *An. coustani* (*n* = 4), and *An. squamosus* (*n* = 4) specimens, to study the aforementioned aims, in addition to *Anopheles caliginosus* (*n* = 1), *An. rufipes* (*n* = 1), and unknown (i.e. unidentified) specimens (*n* = 19).

The samples from Matutuine district (towns of Bela Vista and Catuane) were collected as adult mosquitoes using human-baited tent traps (period: July to December 2021). Detailed information on collection methods and procedures are described elsewhere [9]. In Manhiça village, mosquitoes from were collected as larvae from aquatic breeding sites in and around the village (March 2020 to January 2021), using a standard dipper [27]. Mosquitoes from both areas were collected as part of ongoing operational research activities, and adults were identified morphologically to species using a stereomicroscope and the keys of Gillies and Coetzee [28].

Morphologically identified *An. gambiae* s.l. samples, and samples that could not be morphologically identified due to missing body parts (most commonly the legs) were molecularly identified using the *An. gambiae* s.l. PCR diagnostic assay [29].

All samples were sequenced (ABI3730XL, Applied Biosystems, USA) at the ribosomal DNA internal transcribed spacer region 2 (ITS2) and/or cytochrome oxidase subunit 1 (CO1) loci towards species determination [22, 23, 30]. Molecular identification was conducted blind to morphological identity to prevent any bias in the analysis. Final species confirmation required high sequence identity (98% or greater) to voucher sequences in multiple databases [22, 23, 31, 32]. CO1 and ITS2 database comparisons for each sample were paired to determine species when either CO1 or ITS2 alone did not produce significant results to voucher sequences. Consensus sequences were manually inspected for insertions, deletions, and repeat regions to ensure these sequence differences did not inflate divergence and decrease identity scores. Consensus sequences of each sequence group were compared (BLASTn) to the NCBI and BOLD databases to identify species [23].

## Results and discussion

A total of 62 (out of 81) mosquitoes were identified morphologically to species. Sequencing of all 81 specimens at the ITS2 and/or CO1 location mapped out to 11 sequence groups (putative species). Of these, five sequence groups had both ITS2 and CO1 sequences that had high coverage and percentage identities to sequences in the NCBI and BOLD databases (Table 1). These included *An. arabiensis* (*n* = 5) (samples also confirmed with PCR), *An. coustani* s.s. (*n* = 4), *An. squamosus* (*n* = 3), *An. rufipes* (*n* = 1), and *An. rivulorum* (*n* = 1). With the exception of correctly identified *An. rufipes* and *An. coustani* specimens, all other specimens could not be identified morphologically and were labelled ‘unknown’.

**Table 1:** Morphological anopheline species identification and sequencing results at the ITS2 and/or CO1 location

Morphological identification (n)	ITS2 identification (Contig number)	CO1 identification (Contig number)	Final identification (n)	Species/complex	Notes
Unknown (5)	*An. arabiensis* (583)	*An. arabiensis*	*An. arabiensis* (5)	*An. gambiae* s.l.	PCR confirmed
*An. coustani* (4)	*An. coustani* (584)	*An. coustani* (593)	*An. coustani* (4)	*An. coustani* s.l.	
*An. ziemanni* (16)^A^ *An. tenebrosus* (1)^a^ ***An. pharoensis *(1)** *Unknown* (3)	*An. cf. coustani 1 isolate AN6* (582)	*An. coustani* (593)	*An. cf. coustani 1 isolate AN6* (21)	*An. coustani* s.l.	
*An. tenebrosus* (13)^a^ ***An. pharoensis *(1)** *Unknown* (5)	*An. cf. coustani 2 isolate AN8* (580)	*An. coustani* (593)	*An. cf. coustani 2 isolate AN8* (19)	*An. coustani* s.l.	
*An. pharoensis* (1)	*An. pharoensis isolate AN9* (590)	*An. pharoensis* (594)	*An. pharoensis* (1)	*An. pharoensis*	
*An. pharoensis* (19) ***An. caliginosus *(1)** *Unknown* (4)	*An. cf. pharoensis isolate AN-3* (581)	*An. pharoensis* (594)	*An. cf. pharoensis isolate AN-3* (24)	*An. cf. pharoensis*	
*An. rufipes* (1)	*An. rufipes* (588)	*An. rufipes* (597)	*An. rufipes* (1)	*An. rufipes*	
Unknown (1)	*An. rivulorum* (592)	*An. rivulorum* (599)	*An. rivulorum* (1)	*An. rivulorum*	
Unknown (3)	*An. squamosus* (585)	*An. squamosus* (595)	*An. squamosus* (3)	*An. squamosus*	
***An. squamosus *(1)**	*An. sp. 16 BSL-2014* (587)	*An. sp. 15 JEF-2020 isolate FLMa01407* (596)	Unknown (1)	Unknown	
Unknown (1)	*An. gabonensis* (589)	*An. superpictus* (598)	Unknown (1)	Unknown	Low similarity

Specimens that are misidentified by microscopy are bolded. Sequences for each location can be found in Additional file 1, using the Contig numbers provided in this table

^a^Member of the *An. coustani* group

*Anopheles arabiensis* is historically known to transmit malaria in southern Mozambique [6, 8]. It may currently play a proportionally more significant role in local malaria transmission [33], since *An. funestus* s.s., which was the dominant vector in indoor mosquito collections [4, 34, 35], virtually disappeared after the onset of IRS in the region [8]. *An arabiensis* in the region tends to have higher capturing densities outdoors [9], which is typical for this species [36, 37], thereby reducing the overall efficacy of indoor functioning LLINs. When feeding indoors, it feeds primarily at times when people are in bed, hence increasing net use could significantly reduce the exposure to this vector indoors [33]. It remains unclear if IRS effectively targets this vector, as the majority of data collected demonstrates that this species may be entering and leaving houses without resting on the sprayed surfaces [9]—indicating house entry with undetermined resting behaviour prior to the morning time point of mosquito collections. As argued before [9], hourly indoor aspirations throughout the night would enable the evaluation of any resting behaviour towards understanding the potential impact of IRS.

*Anopeheles squamosus* has been incriminated as a potential secondary vector in southern Mozambique [8] and in neighbouring Zambia [38]. Detailed information on its feeding and resting behaviour are lacking, but it may be highly anthropophilic [14] and, therefore, susceptible to LLINs if feeding indoors.

Though *An. rufipes* has been associated with malaria cases in southern Mozambique based on its vector status and its presence in malaria endemic areas [8], it has not yet been found positive for *Plasmodium falciparum* sporozoites. It is known as a secondary malaria vector in several countries in sub-Saharan Africa [23, 39], and demonstrates typical exophagic and zoophagic behaviours [40], which means indoor vector control may not effectively target this species.

*Anopheles rivulorum* has not been incriminated as a vector in Mozambique, but is a known vector elsewhere in Africa, specifically in the eastern African region [12, 13, 22]. It appears largely zoophilic [40] and may therefore elude indoor vector control.

Two additional sequence groups mapped to the *An. coustani* complex, and included (database placeholder names of) *Anopheles cf. coustani 1* isolate AN6 (*n* = 21), and *An. cf. coustani 2* isolate *AN8* (*n* = 19). Both these sequences (both ITS2 and CO2) have been described before from Kenya and Zambia [22, 23] and represent species in the *An. coustani* complex. These were morphologically identified as either *An. tenebrosus*, *An. ziemanni* (both members of the *An. coustani* group [41]), or unknown—while two were misidentified as *An. pharoensis*.

*Anopheles coustani* s.l. and *An*. *ziemanni* have been incriminated as vectors outside of Mozambique [14–16, 22], whereas *An. tenebrosus* was found positive for *P. falciparum* sporozoites in southern Mozambique [3, 9]. In general, this species group is largely exophagic, and are typically caught in large numbers next to animals [40].

Two sequence groups were similar to *An. pharoensis.* Though the CO1 sequences mapped to this species, the ITS2s were similar to *An. cf. pharoensis* isolate AN3 (*n* = 24) and *An. cf. pharoensis* isolate AN9 (*n* = 1), both also being previously documented in Kenya and Zambia [22, 23]. These were morphologically identified as either *An. pharoensis, An. caliginosus* or unknown. Although *An. pharoensis* has not been incriminated as a malaria vector in Mozambique, it is a known vector elsewhere in sub-Saharan Africa [40, 42]. This species typically demonstrates exophilic and/or exophagic behaviours such that they might elude indoor vector control [40], although there are exceptions to this rule [43, 44].

Two species groups remain unknown (Table 1). One specimen was identified as *Anopheles* sp. 16 BSL-2014 (ITS2, previously documented in Kenya [23]) and *Anopheles* sp. 15 JEF-2020 isolate FLMa01407 (CO1). The other specimen had a very low similarity in the databases with both ITS2 and CO1 sequences, and is given the placeholder name of *Anopheles* sp. 16 MM-2023.

## Conclusions

The aims of this preliminary study were to use molecular identification (CO1 and ITS2 barcoding) to (1) validate the results from the morphological identification (with a particular focus on *An. pharoensis* and *An. squamosus*), and (2) have a closer look at the *An. coustani* group (which includes *An. tenebrosus* and *An. ziemanni*). Out of the 62 specimens that were identified morphologically to species, 4 (or 6.5%) were misidentified. The specific reason(s) for morphological misidentification of these anopheline mosquitoes at this site should be studied further towards its mitigation. It may be related to the capacity of the microscopist to identify anopheline species, but also to the information provided in the dichotomous identification key. This highlights the importance of continuous capacity building in the morphological identification of anopheline species to ensure that malaria control programmes receive timely and accurate data to inform decision-making. This also saves time and money compared to identifying mosquitoes through molecular tools (e.g., PCR, sequencing) in the laboratory [19].

Regarding the *An. coustani* group, morphological identification showed that several members are present in southern Mozambique, including *An. coustani* s.l., *An. ziemanni* and *An. tenebrosus*. However, based on both ITS2 and CO1 sequences, the exact species remains unknown (i.e. they are referred to as *An. coustani* s.l.) until voucher specimens are available for sequencing, or voucher sequences are present in the database. But as this species complex contributes to (residual) malaria transmission in sub-Saharan Africa [22, 23, 40], it could very well contribute to the persistent malaria seen in southern Mozambique. The next step is to analyse a larger subset of mosquitoes that have been collected using a variety of collection methods, to better understand their feeding and resting behaviours as well as their exact role in local malaria transmission. This baseline characterizing of the local anophelines species diversity and behaviours will allow to (a) improve entomological surveillance strategies, (b) better understand the impact of LLINs and IRS on each local vector species, and (c) identify new approaches to target those vector species.

### Supplementary Information

** Additional file 1.** Sequence data.

## Data Availability

All data are available in Table 1 and Additional file 1.
